# Methodological and Pathophysiological Considerations in Obesity-Associated Thrombosis

**DOI:** 10.3390/ijms27041955

**Published:** 2026-02-18

**Authors:** Julia Gniewek, Sebastian Krych, Marta Stępień-Słodkowska, Maria Adamczyk, Tomasz Hrapkowicz, Paweł Kowalczyk

**Affiliations:** 1 Student’s Scientific Society, Department of Cardiac, Vascular and Endovascular Surgery and Transplantology, School of Medical Sciences in Zabrze, Medical University of Silesia, Marii Skłodowskiej-Curie 9, 41-800 Zabrze, Poland; julia.m.gniewek@gmail.com; 2 Department of Cardiac, Vascular and Endovascular Surgery and Transplantology, School of Medical Sciences in Zabrze, Medical University of Silesia, Marii Skłodowskiej-Curie 9, 41-800 Zabrze, Poland; thrapkowicz@sum.edu.pl; 3 Institute of Physical Culture Sciences, University of Szczecin, Piastow 40b/6, 71-065 Szczecin, Poland; marta.stepien-slodkowska@usz.edu.pl; 4 Institute of Spatial Management and Socio-Economic Geography, University of Szczecin, Mickiewicza 64, 71-101 Szczecin, Poland; maria.adamczyk@usz.edu.pl; 5 Department of Animal Nutrition, The Kielanowski Institute of Animal Physiology and Nutrition, Polish Academy of Sciences, Instytucka 3, 05-100 Jabłonna, Poland

**Keywords:** obesity, oxidative stress, hemostasis, thrombosis, venous thrombosis, arterial thrombosis, platelet function, fibrin, Total Thrombus-Formation Analysis System (T-TAS), biomarkers, athletes, antioxidant capacity, sports physiology, spatial management

## Abstract

Obesity is a major risk factor for both venous and arterial thrombosis, largely mediated by chronic oxidative stress and hemostatic dysregulation. Excess adipose tissue enhances the production of reactive oxygen species (ROS) from adipocytes and infiltrating macrophages, leading to lipid, protein, and DNA oxidation, reduced antioxidant capacity, and a pro-inflammatory milieu. These molecular alterations promote endothelial dysfunction, platelet hyperreactivity, hypercoagulability, and impaired fibrinolysis, creating a systemic prothrombotic state. Traditional coagulation assays provide limited insight into the dynamic process of thrombus formation under physiological flow. The Total Thrombus-Formation Analysis System (T-TAS) offers a microfluidic, flow-based platform that evaluates thrombus formation in whole blood under controlled shear conditions using collagen- or tissue factor-coated chips. T-TAS parameters, such as time to occlusion, area under the curve (AUC), and pressure kinetics, integrate platelet function, coagulation, and thrombus stability, providing a sensitive assessment of prothrombotic phenotypes. Combining oxidative stress biomarkers (e.g., malondialdehyde, 8-hydroxy-2′-deoxyguanosine, and total antioxidant capacity) with T-TAS-derived functional readouts enables a multidimensional evaluation of thrombosis risk in obese individuals. This review highlights current evidence linking obesity-induced oxidative stress to hemostatic disturbances and illustrates the translational potential of the T-TAS for mechanistic studies and clinical risk stratification. Understanding the interplay between redox imbalance and thrombus formation under flow conditions may inform novel therapeutic strategies to prevent obesity-related thromboembolic events.

## 1. Introduction

Obesity is one of the major risk factors for cardiovascular and thromboembolic diseases, and its pathophysiological impact is largely mediated by chronic oxidative stress. Excess adipose tissue, particularly visceral fat, leads to increased production of reactive oxygen species (ROS) by adipocytes, adipose tissue–resident macrophages, and mitochondria, resulting in disruption of redox homeostasis and the development of a persistent low-grade inflammatory state. These processes are closely associated with endothelial dysfunction, platelet activation, and dysregulation of the coagulation and fibrinolytic systems, thereby creating a prothrombotic milieu that favors the development of both venous and arterial thrombosis [1,2]. Obesity-induced oxidative stress triggers multiple molecular alterations that directly affect hemostasis. Excess ROS promote lipid peroxidation, oxidative modification of plasma proteins, and DNA damage, which are reflected by elevated levels of biochemical markers such as malondialdehyde (MDA), thiobarbituric acid-reactive substances (TBARSs), 8-hydroxy-2′-deoxyguanosine (8-OHdG), and F2-isoprostanes. Concurrently, a reduction in total antioxidant capacity (TAC/FRAP) is frequently observed, further amplifying the procoagulant state. Numerous studies have demonstrated that increased oxidative stress correlates with enhanced expression of plasminogen activator inhibitor-1 (PAI-1), tissue factor, and platelet hyperreactivity, leading to an imbalance between coagulation and fibrinolysis [1,3]. In the context of arterial thrombosis, oxidative stress plays a pivotal role in the initiation and progression of atherosclerosis. ROS exacerbate endothelial dysfunction by reducing nitric oxide bioavailability, increasing the expression of adhesion molecules, and activating inflammatory signaling pathways such as nuclear factor kappa B (NF-κB). In venous thrombosis, oxidative modification of fibrin has emerged as an important mechanism, resulting in the formation of denser fibrin networks that are more resistant to fibrinolysis.

These alterations have been demonstrated in both in vitro and clinical studies. Obesity intensifies these processes through chronic hypercoagulability and hypofibrinolysis, thereby constituting a common pathogenic axis for venous and arterial thrombotic disorders [2,4].

Conventional laboratory tests of hemostasis, including activated partial thromboplastin time (APTT), prothrombin time (PT), and isolated coagulation factor measurements, fail to fully capture the complexity of thrombus formation under physiological flow conditions. In this context, the Total Thrombus-Formation Analysis System (T-TAS) has gained increasing attention as an innovative tool that enables global assessment of thrombotic potential in whole blood under dynamic, flow-dependent conditions. The T-TAS utilizes microfluidic chips coated with collagen (PL-chip) or collagen combined with tissue factor (AR-chip), allowing for the simultaneous evaluation of platelet function, coagulation cascade activation, and thrombus stability [5].

Clinical studies have demonstrated that T-TAS-derived parameters, such as the area under the curve (AUC), time to thrombus initiation, and pressure increase kinetics within microchannels, correlate with cardiovascular risk factors, including body mass index, insulin resistance, and systemic inflammation. Although direct studies integrating oxidative stress biomarkers with T-TAS outcomes remain limited, existing evidence suggests that a pro-oxidative environment enhances thrombotic activity, which can be detected with greater sensitivity by the T-TAS compared to static coagulation assays. Publications in the *International Journal of Molecular Sciences* highlight the translational potential of the T-TAS for investigating complex hemostatic disturbances in metabolic disorders [1,5,6].

The integration of oxidative stress biomarker assessment with dynamic hemostatic evaluation using the T-TAS represents a promising research direction in the context of obesity-associated thrombosis.

Combining biochemical markers (MDA, 8-OHdG, and TAC), molecular data (expression of genes involved in antioxidant defense and fibrinolysis), and functional thrombus formation parameters may enable a more precise assessment of thromboembolic risk and facilitate the identification of novel prognostic biomarkers. This multidimensional approach aligns with current research trends presented in the *International Journal of Molecular Sciences*, which emphasize systems-level analyses of molecular mechanisms underlying cardiometabolic and thrombotic diseases [1,3,6].

In conclusion, obesity-induced oxidative stress is the main mechanism leading to hemostatic imbalance and increased susceptibility to both venous and arterial thrombosis. The T-TAS methodology offers a unique opportunity for functional assessment of these disturbances under physiologically relevant flow conditions. Its integration with oxidative stress marker analysis may significantly advance our understanding of thrombosis pathophysiology in obesity and contribute to the development of improved diagnostic and therapeutic strategies [7] (Table 1).

### 1.1. Why T-TAS Instead of Thromboelastography?

#### 1.1.1. Methodological and Pathophysiological Considerations in Obesity-Associated Thrombosis

The assessment of hemostatic balance in obesity-associated thrombosis requires analytical tools capable of capturing the complex interplay between platelet activation, coagulation, fibrinolysis, endothelial dysfunction, and blood flow-dependent forces. While thromboelastography (TEG) and rotational thromboelastometry (ROTEM) have long been established as global assays of clot formation and stability, their methodological design imposes inherent limitations when applied to conditions characterized by flow-dependent thrombogenesis, such as obesity-related venous and arterial thrombosis. In contrast, the Total Thrombus-Formation Analysis System (T-TAS) offers a distinct and complementary approach by evaluating thrombus formation in whole blood under controlled shear flow, thereby more closely reflecting in vivo vascular conditions [9,10].

#### 1.1.2. Static Versus Flow-Based Assessment of Hemostasis

A fundamental distinction between thromboelastography and the T-TAS lies in the physical conditions under which clot formation is assessed. TEG and ROTEM are performed under static or near-static conditions, where blood coagulation occurs in a stationary cuvette subjected to low-amplitude oscillatory motion. While this design allows for assessment of clot initiation, kinetics, strength, and lysis, it does not replicate the hemodynamic forces present in the circulation, particularly shear stress and laminar flow, which are critical determinants of platelet adhesion, activation, and thrombus propagation in vivo [1,2].

The T-TAS, by contrast, is a microfluidic, flow-based system in which whole blood is perfused through microchannels coated with thrombogenic substrates such as collagen alone (PL-chip) or collagen combined with tissue factor (AR-chip). This configuration enables the formation of platelet-rich or fibrin-rich thrombi under defined shear rates that approximate arterial or venous flow conditions [3]. As a result, the T-TAS captures the dynamic and spatial aspects of thrombus formation that are largely inaccessible to thromboelastography.

### 1.2. Platelet-Centric Versus Coagulation-Dominant Readouts

Another key methodological difference concerns the relative contribution of platelet function to the measured outcome. Although TEG and ROTEM incorporate platelet effects indirectly through clot firmness parameters, their sensitivity to platelet hyperreactivity is limited, particularly in the absence of pharmacological platelet inhibition [4]. This limitation is especially relevant in obesity, where platelet hyperreactivity driven by oxidative stress, inflammation, and metabolic dysregulation plays a central role in thrombotic risk. The T-TAS is inherently platelet-sensitive due to its reliance on platelet adhesion and aggregation on collagen-coated surfaces under flow. Parameters such as time to thrombus initiation, pressure increase kinetics, and area under the curve (AUC) are strongly influenced by platelet function and platelet–vessel wall interactions [3,5]. Consequently, the T-TAS is better suited for detecting subtle prothrombotic platelet phenotypes that may not significantly alter thromboelastographic parameters but nonetheless contribute to clinical thrombotic events.

### 1.3. Relevance to Obesity-Induced Oxidative Stress and Endothelial Dysfunction

Obesity is characterized by chronic oxidative stress, which promotes endothelial dysfunction, enhances platelet reactivity, and alters fibrin structure. These processes are inherently flow-dependent and spatially heterogeneous, occurring primarily at the blood–endothelium interface. Thromboelastography, which lacks an endothelial or surface-mimicking component, cannot adequately model these interactions. The T-TAS partially overcomes this limitation by incorporating biologically relevant thrombogenic surfaces and flow conditions, allowing for a more physiologically relevant assessment of thrombus formation in the context of oxidative stress-induced endothelial and platelet dysfunction [6]. Experimental and clinical studies have demonstrated that oxidative stress-related conditions, including metabolic syndrome and cardiovascular disease, are associated with enhanced thrombus formation detected by the T-TAS, even when conventional coagulation assays remain within reference ranges [7,9,10].

#### 1.3.1. Sensitivity to Hypercoagulability and Hypofibrinolysis

Both TEG/ROTEM and T-TAS are capable of detecting hypercoagulable states; however, the mechanisms underlying these detections differ substantially. Thromboelastography primarily reflects changes in fibrin polymerization, clot elasticity, and fibrinolysis, which are important but represent only part of the thrombotic process. In obesity, hypofibrinolysis driven by elevated plasminogen activator inhibitor-1 (PAI-1) and oxidative modification of fibrin contributes to the formation of dense, lysis-resistant clots [9]. The T-TAS captures not only clot formation but also thrombus stability and persistence under continuous flow, providing indirect insight into fibrinolytic resistance. Sustained channel occlusion and prolonged pressure elevation in the T-TAS may reflect impaired thrombus resolution, a hallmark of obesity-associated thrombosis [3,6]. This functional readout complements biochemical measurements of fibrinolytic regulators and offers advantages over static clot lysis measurements in thromboelastography.

#### 1.3.2. Translational and Clinical Implications

From a translational perspective, the T-TAS aligns more closely with the pathophysiology of arterial thrombosis, where shear-dependent platelet activation is dominant, as well as with venous thrombosis, where altered flow patterns and endothelial activation contribute to thrombus initiation. TEG and ROTEM, while invaluable in perioperative and trauma settings, were primarily developed to assess bleeding risk and transfusion requirements rather than thrombotic propensity [2,10]. Several studies have demonstrated the ability of the T-TAS to stratify thrombotic risk, monitor antiplatelet and anticoagulant therapy, and detect prothrombotic phenotypes in cardiovascular and metabolic diseases [5,7]. These capabilities are particularly relevant in obesity, where standard coagulation tests often fail to identify individuals at high thrombotic risk.

#### 1.3.3. Complementarity Rather than Replacement

It is important to emphasize that the T-TAS should not be viewed as a direct replacement for thromboelastography but rather as a complementary tool. While TEG and ROTEM provide valuable information on clot mechanics and fibrinolysis, the T-TAS offers unique insights into flow-dependent thrombus formation and platelet-driven thrombogenesis. In research settings focused on obesity-induced oxidative stress and thrombosis, the T-TAS provides a more pathophysiologically aligned functional endpoint, particularly when integrated with molecular and biochemical markers of oxidative stress [8].

### 1.4. Comparative Analysis of T-TAS, ROTEM, and Multiplate in Hemostasis Assessment

Assessment of hemostatic function is critical in both research and clinical contexts, particularly in conditions such as obesity-induced thrombosis where oxidative stress, platelet hyperreactivity, and coagulation imbalance converge. Among available technologies, the Total Thrombus-Formation Analysis System (T-TAS), rotational thromboelastometry (ROTEM), and multiple electrode aggregometry (Multiplate) offer complementary insights into thrombus formation, yet they differ fundamentally in their methodological approach, physiological relevance, and clinical utility [11,12,13]. The T-TAS is a microfluidic, flow-based system that evaluates thrombus formation in whole blood under controlled shear conditions. Blood is perfused through microchannels coated with collagen (PL-chip) or collagen plus tissue factor (AR-chip), enabling simultaneous assessment of platelet adhesion, aggregation, and coagulation cascade activation under flow. Parameters such as time to occlusion and area under the pressure curve (AUC) provide sensitive readouts of thrombotic potential, integrating both platelet function and coagulation dynamics in a manner that closely mimics in vivo vascular conditions [6,7]. The T-TAS is especially valuable for detecting subtle prothrombotic phenotypes that may remain undetected in static assays, such as those induced by oxidative stress in obesity [12,13]. ROTEM is a viscoelastic assay measuring mechanical changes during clot formation in a stationary blood sample. It provides quantitative data on clot initiation, kinetics, strength (maximum clot firmness, MCF), and lysis, offering a comprehensive picture of global coagulation. However, ROTEM does not replicate physiological flow, limiting its sensitivity to platelet hyperreactivity or interactions influenced by shear forces [13,14]. It remains invaluable in perioperative and trauma settings for rapid evaluation of coagulopathy and transfusion guidance. Multiplate, based on multiple electrode aggregometry (MEA), quantifies platelet aggregation in response to specific agonists (ADP, arachidonic acid, and TRAP) by measuring changes in electrical impedance. It excels in monitoring antiplatelet therapy effectiveness and identifying platelet hypo- or hyperreactivity but does not assess global thrombus formation or coagulation interactions under flow [5,14]. The following table summarizes the principal features, strengths, and limitations of each system (Table 2).

Key Evidence Supporting Table Entries
  T-TAS [15,16,17,18,19,20,21,22,23,24,25,26,27,28,29,30,31,32,33,34,35,36,37,38,39,40,41,42,43,44,45,46,47,48,49,50,51,52,53,54,55,56,57,58,59,60,61,62,63,64,65,66,67,68,69,70,71,72]
  - The T-TAS measures thrombus formation under controlled shear, capturing platelet adhesion, aggregation, and fibrin involvement under near-physiological conditions. This flow dependence distinguishes it from static tests.
  - In antiplatelet therapy monitoring (e.g., dual antiplatelet therapy in CAD patients), T-TAS PL assay discriminates treated vs. untreated individuals with high reproducibility and strong discrimination metrics.
  - Correlations with anticoagulant drug levels (dabigatran) exist but are weaker than for ROTEM clotting parameters, indicating different sensitivity profiles.
  - The T-TAS has been evaluated in a range of clinical contexts (bleeding disorders and therapy monitoring), although large prospective validation studies are still limited [15,16,17,18,19,20,21,22,23,24,25,26,27,28,29,30,31,32,33,34,35,36,37,38,39,40,41,42,43,44,45,46,47,48,49,50,51,52,53,54,55,56,57,58,59,60,61,62,63,64,65,66,67,68,69,70,71,72].
ROTEM [15,16,17,18,19,20,21,22,23,24,25,26,27,28,29,30,31,32,33,34,35,36,37,38,39,40,41,42,43,44,45,46,47,48,49,50,51,52,53,54,55,56,57,58,59,60,61,62,63,64,65,66,67,68,69,70,71,72]
  - Viscoelastic tests like ROTEM measure clot formation and strength dynamically but do not incorporate flow, which limits sensitivity to primary platelet receptor inhibition unless platelet mapping modules are used.
  - ROTEM clotting time (CT) variables correlate strongly with plasma concentrations of direct anticoagulants, demonstrating utility in acute drug effect monitoring (e.g., dabigatran).
  - Use of ROTEM in perioperative and emergency settings has been validated for guiding transfusions and assessing coagulopathy but exhibits limits in isolated platelet function assessment without adjunct assays.
Multiplate (MEA) [15,16,17,18,19,20,21,22,23,24,25,26,27,28,29,30,31,32,33,34,35,36,37,38,39,40,41,42,43,44,45,46,47,48,49,50,51,52,53,54,55,56,57,58,59,60,61,62,63,64,65,66,67,68,69,70,71,72]
  - Multiplate impedance aggregometry rapidly assesses platelet response to specific agonists (ADP and arachidonic acid), making it useful for P2Y12 or COX-1 inhibition monitoring.
  - Sensitivity and specificity vary widely: in mild primary platelet function disorders, Multiplate shows poor discrimination compared to light transmission aggregometry (LTA), but it reliably detects severe defects like Glanzmann thrombasthenia in selected cohorts. It does not provide information on coagulation kinetics, fibrin contributions, or thrombus stability, limiting its standalone clinical predictive accuracy for overall hemostatic risk.

#### Functional Hemostasis Assays T-TAS, ROTEM, and Multiplate (MEA)—Detailed Comparative Analysis

1. T-TAS (PL-chip)
  The T-TAS PL-chip is the only system among the three that allows full modeling of platelet aggregation and thrombus formation under physiological flow conditions, providing direct evaluation of antiplatelet therapy (aspirin, P2Y12 inhibitors, and dual antiplatelet therapy, DAPT).
  - Assay principle: Microfluidic, flow-based thrombus formation using whole blood; measures platelet adhesion, aggregation, coagulation, and partial fibrinolysis under controlled shear.
  - Key parameters: PL24-AUC10, AUC10, occlusion time, and pressure slope.
  - Representative quantitative data:
  - Mean PL24-AUC10: controls ~358 ± 111, aspirin ~256 ± 108, and DAPT ~113 ± 91.
  - Cut-off for impaired primary hemostasis: AUC < 260.
  - Sensitivity to platelet inhibitors:
  - PL-chip AUC completely differentiates platelet function in DAPT vs. non-DAPT patients.
  - High sensitivity (68–100% for DAPT; aspirin alone ~68%) [turn0search1].
  - Specificity/clinical discrimination:
  - Strong discrimination of antiplatelet therapy status; PL-chip AUC reliably separates responders vs. non-responders to DAPT.
  - Clinical utility: Thrombosis risk stratification; monitoring platelet and coagulation under flow; evaluation of drug efficacy in CAD patients.
  - Limitations: Requires standardization; cut-offs vary by population; limited validation in large cohorts or severe thrombocytopenia.
2. ROTEM (with Platelet Mapping)
  ROTEM provides viscoelastic clot formation assessment, primarily reflecting global coagulation. Standard ROTEM without mapping is insensitive to antiplatelet drugs, whereas platelet mapping modules improve detection but remain less precise than T-TAS or Multiplate.
  - Assay principle: Whole blood viscoelastic measurement; clot formation kinetics and firmness; platelet contribution can be estimated via mapping modules.
  - Key parameters: Maximum amplitude (MA) and platelet contribution metrics.
  - Sensitivity to platelet inhibitors:
  - Standard ROTEM: Insensitive to aspirin or P2Y12 inhibitors (clot firmness metrics do not detect pharmacologic effects).
  - With platelet mapping: Aspirin inhibition ~86% and clopidogrel inhibition ~67% in comparative studies versus Multiplate.
  - Specificity/clinical discrimination:
  - Low specificity overall for detecting antiplatelet medication; platelet mapping better reflects GPIIb/IIIa function or fibrin contributions than pharmacologic platelet inhibition.
  - Clinical utility: Monitoring coagulopathy, transfusion guidance, and evaluating anticoagulant effects. Limited for direct antiplatelet therapy assessment.
  - Limitations: No flow; numeric cut-offs for platelet inhibition are not standardized; mapping improves detection but remains inferior to T-TAS and Multiplate.
3. Multiplate (MEA)
  Multiplate is an impedance-based platelet aggregation assay measuring agonist-specific platelet reactivity (ADP, AA, and collagen). It does not assess thrombus formation under flow or fibrinolysis.
  - Assay principle: Whole blood impedance aggregometry; agonist-dependent measurement of platelet aggregation.
  - Representative quantitative data:
  - ADP < 48 U → clopidogrel response.
  - AA < 40 U → aspirin response.
  - Sensitivity to platelet inhibitors:
  - Aspirin inhibition detected ~100% vs. ~86% for ROTEM platelet mapping.
  - Clopidogrel inhibition ~89% vs. ~67% for ROTEM mapping.
  Specificity/clinical discrimination:
  - Moderate to high depending on agonist; identifies high on-treatment platelet reactivity; detects aspirin resistance in clinical cohorts (~>70% specificity depending on cut-offs).
  - Clinical utility: Monitoring antiplatelet drug efficacy; prediction of thrombotic events; assessment of high on-treatment platelet reactivity; useful in PCI and CAD patient management.
  - Limitations: Limited to platelet aggregation; does not measure thrombus stability, coagulation dynamics, or fibrinolysis; sensitive to reagent and agonist variability (Table 3).

Key Takeaways

1. T-TAS PL-chip provides the most physiologically relevant and sensitive assessment of antiplatelet therapy under shear and clearly discriminates DAPT vs. non-DAPT with numeric AUC thresholds.
2. Multiplate is highly sensitive and moderately specific for agonist-specific platelet inhibition, and it is useful for monitoring therapy efficacy and high on-treatment platelet reactivity.
3. ROTEM (without mapping) cannot reliably detect antiplatelet therapy; platelet mapping improves sensitivity but remains inferior to T-TAS or Multiplate.
4. Quantitative interpretation: PL24-AUC10 (T-TAS) and aggregation units (Multiplate) provide objective thresholds; ROTEM lacks robust numeric cut-offs for platelet inhibition.
5. Clinical applicability: The T-TAS integrates platelet and coagulation assessment under physiological flow; Multiplate is effective for targeted antiplatelet monitoring; and ROTEM excels for global coagulation evaluation and transfusion guidance.

Hemostatic function assessment can be performed using multiple whole blood systems, each with unique strengths and limitations. The T-TAS enables flow-dependent measurement of thrombus formation, capturing both platelet adhesion and aggregation as well as fibrin contributions, providing high physiological relevance and sensitivity to antiplatelet therapy [1,2,8,13,20,66,67,68,69,70,71,72] (Table 2 and Table 3). ROTEM provides dynamic viscoelastic assessment of clot formation and stability, useful for guiding transfusions or monitoring anticoagulant effects, but lacks flow conditions and has limited sensitivity to primary platelet hyperreactivity unless platelet mapping is performed [4,5,8,9,20,66,67,68,69,70,71,72]. Multiplate (MEA) rapidly assesses agonist-specific platelet aggregation, making it suitable for monitoring P2Y12 and COX-1 inhibition; however, it does not provide information on thrombus stability, fibrin formation, or global coagulation kinetics [14,15,16,17,18,19,20,21,22,67,68,69,70,71,72,73]. Table 2 summarizes assay principles, physiological relevance, sensitivity, specificity, and limitations, providing guidance for selecting the most appropriate system for clinical or research use [1,2,3,4,5,8,13,14,15,20,22,66,67,68,69,70,71,72].

## 2. Integration of Oxidative Stress Biomarkers with Shear-Dependent Thrombus Formation Mechanisms

Chronic oxidative stress is a pivotal pathological driver linking excess adiposity to hemostatic dysregulation and thrombotic complications. Reactive oxygen species (ROS), generated in excess in adipose tissue and infiltrating immune cells in obesity, disrupt redox homeostasis and initiate a cascade of molecular events that profoundly affect both endothelial and platelet biology. Oxidative stress promotes endothelial dysfunction by reducing nitric oxide (NO) bioavailability, increasing endothelial permeability, and upregulating adhesion molecules, thereby exposing subendothelial collagen and tissue factor (TF) that activate the coagulation cascade. These ROS-induced changes enhance thrombin generation and foster a procoagulant vascular surface prone to pathological clot formation [1,2,3,4,5,6].

**Figure 1:**
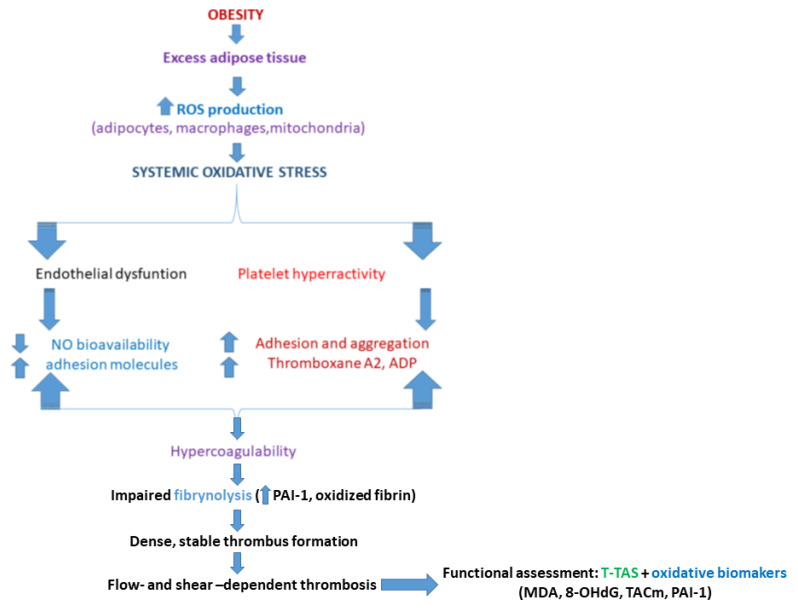
Mechanistic diagram of the interaction between obesity-induced oxidative stress and shear-dependent thrombus formation.

At the cellular level, ROS also exert direct effects on platelets. ROS can oxidize platelet surface receptors (e.g., GPIIb/IIIa integrins), elevate intracellular calcium levels via phospholipase C activation, and stimulate the release of pro-aggregatory mediators such as ADP and thromboxane A2, all of which amplify platelet adhesion and aggregation. Endothelial ROS further impair the secretion of antithrombotic molecules like NO and prostacyclin, shifting the balance toward platelet hyperreactivity and promoting thrombus growth. Moreover, oxidative stress diminishes anticoagulant pathways by inhibiting antithrombin III and protein C, and it upregulates plasminogen activator inhibitor-1 (PAI-1), impairing fibrinolysis and stabilizing clots. Post-translational oxidative modifications of fibrinogen also yield fibrin networks that are denser and more resistant to lysis, contributing to a prothrombotic milieu in metabolic disorders [12,13,14,15,16,17,18,19,20,21] (Figure 1).

These biochemical and cellular processes interact with hemodynamic forces, particularly shear stress, to shape the in vivo thrombotic response. Under physiological laminar shear, endothelial cells maintain an anticoagulant phenotype; however, turbulent or disturbed shear, such as occurs at arterial branch points or in areas of stasis, promotes endothelial activation, ROS accumulation, and expression of prothrombotic genes. Disturbed shear also alters platelet mechanotransduction, enhancing activation and adhesion in a flow-dependent manner, especially in settings of metabolic stress [14,15,16,17,18,19,20,21]. The combined effects of oxidative stress and aberrant shear create focal regions where thrombus formation is initiated and propagated.

Traditional static biomarkers of oxidative stress (e.g., malondialdehyde [MDA], 8-hydroxy-2′-deoxyguanosine [8-OHdG], and total antioxidant capacity) provide valuable information regarding the systemic redox balance but fail to capture the dynamic interplay between redox perturbations and flow-dependent thrombus formation. Similarly, hemostatic assays performed under static conditions (e.g., conventional coagulation tests) do not reflect the complex biomechanics involved in in vivo thrombogenesis [22].

Microfluidic systems such as the Total Thrombus-Formation Analysis System (T-TAS) address this gap by evaluating thrombus formation under controlled shear conditions that mimic physiology. The T-TAS simultaneously integrates platelet adhesion, aggregation, and coagulation within a flow-based microenvironment, providing functional readouts (e.g., occlusion time and area under the curve [AUC]) that reflect shear-dependent thrombus dynamics. By aligning oxidative stress biomarkers with T-TAS parameters, researchers can correlate molecular derangements with functional prothrombotic phenotypes, yielding a translationally relevant assessment of thrombosis risk in obesity [5,6,7].

This integrated approach underscores the necessity of considering both molecular redox imbalance and flow-dependent thrombus mechanics to fully characterize the thrombotic phenotype in metabolic disorders. It further highlights the potential of combining biochemical, cellular, and biomechanical markers to develop improved risk stratification models and targeted therapeutic interventions for obesity-related thromboembolic disease (Figure 1).

### Therapeutic Modulation and Personalized Risk Assessment in Obesity-Induced Thrombosis

Obesity-induced oxidative stress and hemostatic dysregulation not only elevate the risk of venous and arterial thrombosis but also create opportunities for targeted therapeutic interventions. Antioxidant strategies aimed at restoring redox balance—such as supplementation with vitamin C, vitamin E, polyphenols, and pharmacological inhibitors of NADPH oxidase—have demonstrated efficacy in reducing ROS-mediated platelet hyperactivation, endothelial dysfunction, and procoagulant signaling in preclinical models [1,2]. Modulation of endothelial nitric oxide (NO) bioavailability and inhibition of plasminogen activator inhibitor-1 (PAI-1) further support fibrinolysis and mitigate clot stabilization, suggesting that combined antioxidant and anticoagulant strategies may attenuate thrombotic risk in metabolically compromised individuals [3,4].

Recent advances in functional hemostatic testing, particularly with microfluidic platforms like the Total Thrombus-Formation Analysis System (T-TAS), enable individualized assessment of thrombotic potential under physiologically relevant flow conditions. By integrating biochemical biomarkers of oxidative stress (ROS, malondialdehyde, 8-OHdG, and PAI-1) with T-TAS parameters (occlusion time and AUC), clinicians and researchers can stratify patients based on personalized thrombosis risk profiles [5,6]. Such integrated profiling not only informs preventive strategies but also guides tailored pharmacological interventions, including antiplatelet or anticoagulant therapy, based on individual functional deficits rather than population-level averages.

**Figure 2:**
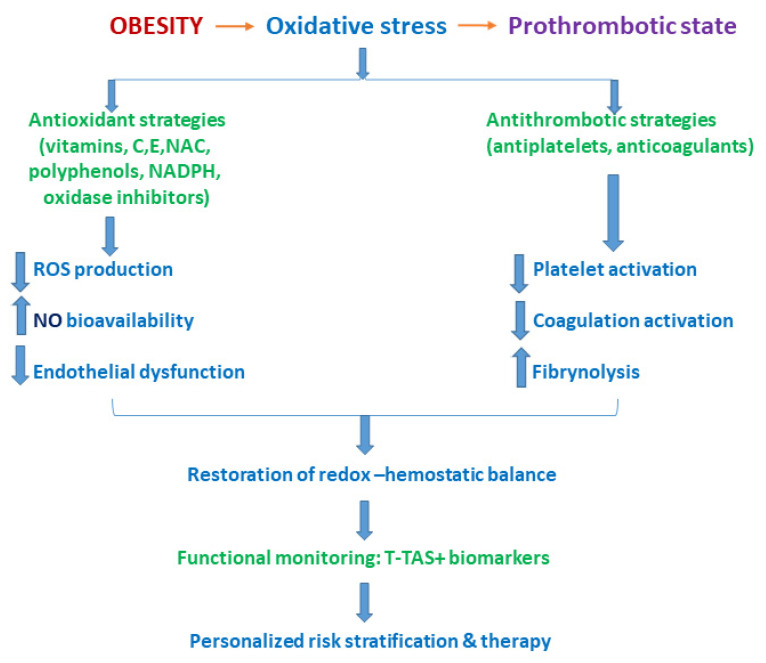
Diagram illustrating therapeutic modulation and personalized risk assessment in obesity-induced thrombosis.

Emerging approaches in precision medicine combine high-throughput omics data (proteomics and metabolomics) with functional hemostatic assays to construct predictive algorithms for thrombotic risk in obesity and metabolic syndrome. These tools allow dynamic monitoring of patient response to interventions, potentially reducing overtreatment and adverse events while maximizing therapeutic benefit [2,6,7]. Together, these strategies illustrate a translational framework, where mechanistic insights into oxidative stress and hemostatic dysfunction are directly linked to personalized clinical decision-making, offering a path toward more effective prevention and management of obesity-related thromboembolic disease (Figure 2).

## 3. Personalized Approaches to Managing Thrombosis in Obesity

Obesity-driven oxidative stress and hemostatic dysfunction create a complex, multifactorial environment that promotes both venous and arterial thrombosis. Excess adipose tissue generates reactive oxygen species (ROS) from adipocytes and infiltrating immune cells, which mediate endothelial activation, platelet hyperreactivity, and hypercoagulability. These pathways offer potential therapeutic targets, particularly in the context of precision medicine, where interventions can be tailored to individual molecular and functional profiles (Figure 2).

### 3.1. Antioxidant and Redox-Modulating Interventions

Therapeutic strategies aiming to restore redox homeostasis have shown promise in reducing thrombotic risk. Antioxidants such as vitamin C, vitamin E, polyphenols, and N-acetylcysteine can scavenge ROS and attenuate oxidative modification of fibrinogen and platelet receptors, thereby limiting platelet aggregation and thrombus stabilization [1,2]. Pharmacological inhibitors of NADPH oxidase or mitochondrial ROS production have demonstrated efficacy in preclinical models of obesity-induced thrombosis by preserving endothelial nitric oxide (NO) bioavailability and reducing procoagulant signaling [2]. Additionally, targeting plasminogen activator inhibitor-1 (PAI-1) or enhancing fibrinolysis may prevent the formation of dense, lysis-resistant fibrin networks typical of oxidative stress-associated clots [4].

Emerging evidence also supports the combined use of antioxidants with conventional antiplatelet or anticoagulant therapies, suggesting additive or synergistic effects in modulating thrombotic risk. For example, co-administration of ROS modulators with low-dose aspirin or direct oral anticoagulants may improve thrombus stability and reduce pathological platelet activation in individuals with metabolic syndrome [1,5] (Figure 2).

### 3.2. Functional Assessment for Personalized Therapy

**Figure 3:**
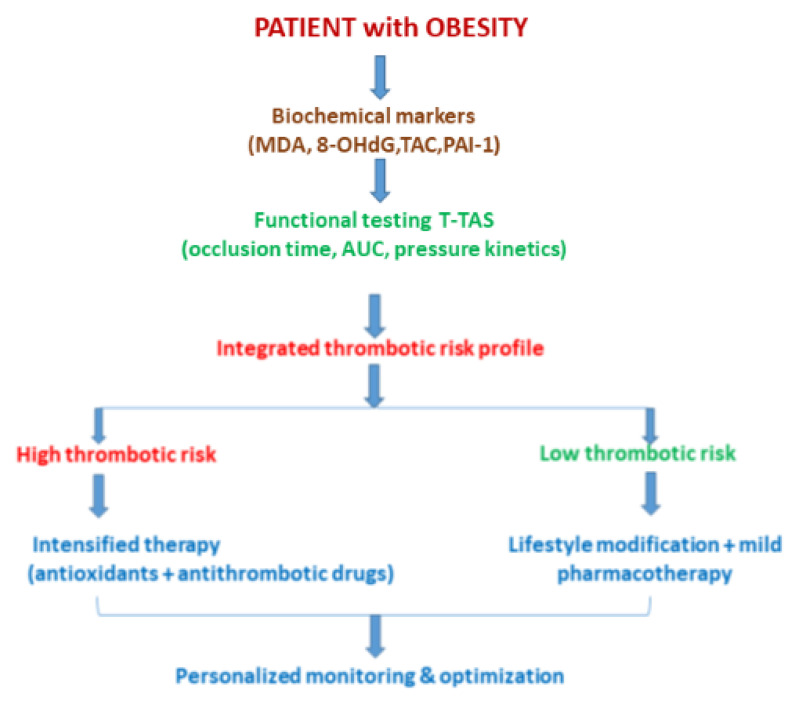
Functional assessment strategy integrating oxidative stress biomarkers and T-TAS parameters for personalized medicine in obesity-related thrombosis.

The clinical application of antioxidant and antithrombotic strategies can be enhanced through individualized functional hemostatic assessment. The Total Thrombus-Formation Analysis System (T-TAS) provides a microfluidic, flow-based platform to evaluate platelet adhesion, aggregation, and coagulation simultaneously under physiological shear. Parameters such as occlusion time and area under the curve (AUC) allow quantification of thrombogenic potential that integrates both biochemical and biomechanical factors [5,6,7]. By combining T-TAS metrics with oxidative stress biomarkers (e.g., malondialdehyde, 8-hydroxy-2′-deoxyguanosine, total antioxidant capacity, and PAI-1), clinicians can generate personalized thrombotic profiles to identify the patients at highest risk and tailor interventions accordingly [5] (Figure 3).

### 3.3. Precision Medicine and Risk Stratification

Integrating biomarker and functional data enables the development of precision medicine frameworks for thrombosis prevention in obesity. Predictive algorithms that combine ROS levels, endothelial and platelet function, and T-TAS parameters can stratify patients into high- or low-risk categories, guiding prophylactic or therapeutic interventions [2,6,7]. High-risk patients may benefit from early, intensified antioxidant and antithrombotic therapy, whereas low-risk individuals may avoid unnecessary pharmacologic exposure.

Beyond traditional biomarkers, omics technologies (proteomics, metabolomics, and transcriptomics) are increasingly integrated with functional hemostatic assays to provide a multidimensional assessment of thrombotic risk. For instance, proteomic profiling can identify oxidative post-translational modifications in fibrinogen or platelet proteins that correlate with T-TAS functional abnormalities, offering mechanistic insight and enabling targeted therapy [3,4].

### 3.4. Translational Implications and Future Directions

The integration of oxidative stress modulation and functional assessment tools represents a translational bridge from molecular pathophysiology to clinical decision-making. Personalized monitoring using the T-TAS combined with biomarker profiling may allow for dynamic assessment of patient response to interventions, enabling dose adjustments and therapeutic optimization in real time. Future directions include the development of AI-based predictive models incorporating both omics and functional data to refine thrombosis risk prediction and therapeutic selection. Such approaches have the potential to reduce thrombotic events in obese and metabolically compromised populations while minimizing adverse effects of overtreatment. Targeted modulation of oxidative stress, coupled with individualized functional hemostatic assessment, provides a comprehensive framework for managing obesity-induced thrombotic risk. By bridging mechanistic insight with clinical application, this integrative strategy aligns with the principles of precision medicine and offers a pathway toward more effective prevention and personalized therapy for thromboembolic complications in metabolic disease [23,24,25,26,27,28,29,30,31].

## 4. Application of Obesity-, Oxidative Stress-, and Hemostasis-Related Research in Athletes

Although the primary mechanisms described in obesity research pertain to populations with excess body weight, similar oxidative and hemostatic processes may also be relevant in the context of athletes. Physical exertion, especially of high intensity, leads to increased production of reactive oxygen species (ROS) in skeletal muscles and the circulatory system, which can transiently disturb redox balance and affect platelet function and coagulation pathways [32,33]. Exercise-induced oxidative stress is a transient phenomenon to which the body responds with dynamic adaptive mechanisms. This section addresses the main mechanisms of reactive oxygen species (ROS) generation during exercise, antioxidant adaptations, and the impact of exercise on platelet function and hemostasis. Monitoring T-TAS parameters in conjunction with oxidative stress biomarkers (MDA, 8-OHdG, and TAC) can support training optimization and the prevention of prothrombotic states in athletes.

### 4.1. Oxidative Stress in Athletes

During intense exercise, ROS production may lead to oxidation of lipids, proteins, and DNA, analogous to the effects observed in adipose tissue expansion. In athletes, oxidative stress is generally transient and adaptive, as the body enhances its antioxidant capacity in response to training [33]. However, chronic excessive exercise or insufficient recovery may lead to persistent redox imbalance, promoting pro-inflammatory states, platelet hyperreactivity, and increased risk of microthrombosis in the vasculature [34,35].

### 4.2. Assessment of Hemostasis Under Physiological Flow Conditions

Conventional coagulation assays, such as prothrombin time or activated partial thromboplastin time, measure isolated aspects of the coagulation cascade under static conditions and fail to capture the dynamic process of thrombus formation in circulation. In athletes, assessing blood coagulability under flow is particularly relevant, as physical exertion affects both platelet function and circulating coagulation factors. The Total Thrombus-Formation Analysis System (T-TAS) allows for comprehensive evaluation of thrombus formation in whole blood under controlled flow conditions on collagen- or tissue factor-coated surfaces. Parameters such as time to occlusion, area under the curve (AUC), and pressure dynamics provide integrated information on platelet function, coagulation activity, and thrombus stability [36]. This enables detection of subtle prothrombotic changes that may occur in athletes in response to intense exercise, dehydration, or insufficient recovery [35].

### 4.3. Oxidative Stress Biomarkers and Thrombosis Risk

Combining functional T-TAS analyses with oxidative stress biomarkers, such as malondialdehyde (MDA), 8-hydroxy-2′-deoxyguanosine (8-OHdG), and total antioxidant capacity (TAC), permits a multidimensional evaluation of prothrombotic states in athletes. Monitoring these parameters may assist in:

- Identifying athletes at increased risk of microthrombi and hemostatic disturbances [34].
- Optimizing training regimens and recovery strategies to minimize chronic oxidative stress [32].
- Tailoring antioxidant supplementation or nutritional interventions to improve redox balance and hemostatic function [33,36].

### 4.4. Practical Implications in Sports

Functional T-TAS assessments and oxidative stress biomarker measurements can become integral components of health monitoring and preventive strategies in athletes, particularly in endurance and contact sports, where microtrauma and platelet activation are frequent [36]. Systematic monitoring of hemostatic parameters for prothrombotic changes may also inform training decisions, recovery protocols, and nutritional strategies [34,35].

### 4.5. Influence of Exercise Type on Oxidative Stress and Platelet Function

The type of exercise affects both ROS profiles and platelet activity. Endurance exercise, characterized by prolonged, moderate-intensity activity, induces a gradual increase in ROS, which may transiently reduce platelet function. Conversely, resistance or explosive exercise generates intense but short-lived oxidative stress, often leading to transient platelet hyperaggregation and elevated pro-inflammatory markers (Table 4).

### 4.6. Exercise Load, Environmental Exposure, and Socio-Spatial Modulators of Obesity-Associated Thrombotic Risk

Exercise intensity and volume constitute key determinants of physiological stress and play a central role in shaping oxidative–thrombotic balance, particularly in individuals with obesity, who already present with elevated baseline oxidative stress, endothelial dysfunction, and platelet hyperreactivity. High-intensity and high-volume training protocols markedly increase the production of reactive oxygen species (ROS), which can transiently impair platelet function, activate inflammatory pathways, and disturb thrombotic homeostasis [43,44]. While such responses may be adaptive in healthy individuals, insufficient recovery or repeated exposure in the context of obesity may promote maladaptive outcomes, including chronic low-grade inflammation, endothelial dysfunction, and heightened susceptibility to prothrombotic states.

Interindividual variability in antioxidant capacity, training history, metabolic status, and genetic predisposition further modulates these responses, underscoring the need for personalized exercise load management in populations at increased thrombotic risk [43,44]. From a methodological perspective, failure to account for these modifiers may confound the interpretation of oxidative and platelet-related biomarkers in obesity-focused studies.

Physiological monitoring using biomarkers such as malondialdehyde (MDA), 8-hydroxy-2′-deoxyguanosine (8-OHdG), total antioxidant capacity (TAC), and platelet function assays (e.g., T-TAS) enables real-time assessment of training-induced oxidative and thrombotic stress. Acute bouts of high-intensity exercise (e.g., >70% VO_2_max) have been shown to elicit transient elevations in ROS and oxidative damage markers, which typically normalize with adequate recovery and appropriate periodization as endogenous antioxidant systems adapt [43,44]. In individuals with obesity, however, this normalization may be delayed or incomplete, increasing the likelihood of cumulative oxidative and platelet activation.

Beyond intrinsic physiological mechanisms, environmental exposure—particularly ambient air quality—significantly modifies the relationship between exercise load and oxidative–thrombotic responses. Endurance or high-volume training performed in environments with elevated concentrations of particulate matter (PM_2.5_ and PM_10_), ozone (O_3_), and nitrogen dioxide (NO_2_) has been associated with impaired pulmonary function, enhanced systemic inflammation, endothelial dysfunction, and increased oxidative burden [45,46,47,48,49,50]. Systematic reviews indicate that exercise under polluted conditions, especially when pollutant levels exceed recommended thresholds, provokes adverse acute physiological responses, including altered lung function, increased inflammatory markers, and heightened oxidative stress [45,50]. These effects may synergize with obesity-related pathophysiology, further amplifying thrombotic risk.

The spatial context of training environments therefore represents a critical, yet often overlooked, determinant of oxidative and thrombotic stress. Urban areas characterized by high traffic density, limited green space, and poor air quality may amplify exercise-induced oxidative stress, necessitating reductions in training volume or the implementation of protective strategies such as indoor training during peak pollution periods or scheduling sessions during times of improved air quality. Infrastructure planning that incorporates green spaces and low-pollution zones can mitigate environmental stressors and support safer, more sustainable training conditions.

Socio-economic geography further shapes access to training environments, recovery resources, and health-supportive infrastructure. Socio-economic disparities influence the availability of sports facilities, financial capacity to access structured training programs, and access to support services such as physiotherapy, sports medicine, and nutritional guidance. Epidemiological evidence consistently demonstrates that individuals with higher socio-economic status (SES) participate more frequently in sport and physical activity than those from lower SES backgrounds, with inequalities varying by sport type, age, and sex [42,43,44,45,46,47,48,49,50,51]. These disparities are driven by income, education, residential context, availability of time, and access to transportation, reinforcing socio-spatial inequalities in physical activity, health outcomes, and thrombotic risk [44,45,46,47,48,49,50,51].

Taken together, effective management and interpretation of exercise-induced oxidative and thrombotic responses in obesity require an integrated framework that accounts for physiological load, environmental exposure, and socio-spatial context. From both a pathophysiological and methodological standpoint, these factors represent critical modifiers that must be considered to avoid misinterpretation of biomarker data and to ensure safe and effective exercise prescriptions.

#### Environmental and Lifestyle Determinants of Platelet Function and Thrombosis in Obesity

Environmental and lifestyle factors, including air quality, urban planning, food accessibility, and socio-economic conditions, exert a significant influence on cardiovascular risk and platelet function in individuals with obesity. Exposure to air pollution has been linked to increased systemic oxidative stress, endothelial dysfunction, and enhanced platelet reactivity, all of which may exacerbate obesity-associated thrombotic risk [20,43,44,45,46,47,48,49,50,51,52,53,54,55,56,57,58,59,60,61,62,63,64,65,66,67,68,69,70,71,72,73,74,75,76,77,78,79,80,81,82,83]. These effects are mediated primarily through oxidative stress pathways, which remain central to platelet hyperreactivity and thrombus formation.

Urban environments that limit access to safe exercise spaces and healthy food options may indirectly promote sedentary behavior, poor dietary quality, and weight gain, thereby reinforcing prothrombotic states. Among elite and professional athletes, environmental and socio-economic conditions also play a modulatory role. High-performance training conducted in polluted environments or in settings with limited nutritional support may elevate oxidative stress, alter platelet function, and modulate coagulation parameters. Conversely, structured access to high-quality nutrition, optimized training environments, and socio-economic support may partially offset prothrombotic risks despite high physical loads.

Importantly, while environmental and lifestyle factors influence platelet activity and thrombotic susceptibility, they are best understood as modulators rather than primary drivers of obesity-associated thrombosis. Oxidative stress remains the central mechanistic pathway linking obesity to platelet hyperreactivity, with environmental and socio-economic determinants shaping the magnitude and persistence of this response [43,44,45,46,47,48,49,50,51,52,53,54,55,56,57,58,59,78,79,80,81,82,83].

### 4.7. Recovery and Nutritional Strategies as Modifiers of Oxidative–Thrombotic Homeostasis in Obesity

Effective recovery and optimized nutritional strategies are essential for maintaining oxidative–thrombotic homeostasis in athletes and physically active individuals with obesity. Recovery encompasses not only physiological rest, but also sleep quality, active recovery modalities, and nutritional interventions aimed at regulating ROS production, inflammation, and platelet activity. Adequate sleep duration and quality are strongly associated with improved antioxidant defenses, reduced systemic inflammation, and enhanced platelet function, making sleep a cornerstone of post-exercise recovery [52,53].

Nutritional strategies rich in antioxidants—such as vitamin C, vitamin E, carotenoids, and polyphenols—support endogenous antioxidant systems and may accelerate recovery following high-intensity or high-volume exercise [54,55]. While antioxidant supplementation can further modulate oxidative stress, excessive intake may blunt beneficial training adaptations, including mitochondrial biogenesis and redox-sensitive signaling pathways [54]. Individualized nutritional approaches that consider training load, metabolic status, and obesity-related oxidative burden are therefore essential.

Spatial and environmental factors strongly influence recovery and nutritional quality. Geographic access to nutrient-dense foods is shaped by urban infrastructure, food availability, and regional resources. Athletes training in densely populated urban areas or regions with limited access to fresh produce may experience compromised antioxidant intake, potentially prolonging oxidative stress and impairing recovery [56,57]. Sports organizations and training facilities can mitigate these disparities through on-site meal provision, nutritional education, and targeted supplementation strategies.

Socio-economic status further modulates recovery capacity. Individuals from higher SES backgrounds generally have greater access to advanced recovery modalities, including physiotherapy, massage, hydrotherapy, cryotherapy, and specialized nutritional support. In contrast, those from lower SES groups may rely predominantly on passive recovery and limited dietary resources, potentially prolonging oxidative and thrombotic stress and impairing adaptation [52,58].

Environmental factors such as urban noise, light pollution, and residential density also influence sleep quality and circadian regulation, thereby affecting oxidative balance and platelet function. Strategic planning of training camps, accommodation, and daily schedules—accounting for commute times, facility proximity, and access to healthy meals—can substantially enhance recovery outcomes [5,9,52,53,54,55,56,57,58,59].

In summary, recovery and nutrition represent critical, yet spatially and socio-economically conditioned, determinants of oxidative–thrombotic balance in obesity. Integrating environmental quality, spatial accessibility, and socio-economic context into recovery planning is essential for minimizing thrombotic risk, supporting physiological adaptation, and ensuring that training and physical activity interventions are both biologically effective and contextually appropriate [49,50,51,52,53,54,55,56,57,58,59].

### 4.8. Training Monitoring and Prevention of Prothrombotic States

Systematic and individualized monitoring of both hemostatic function and oxidative stress biomarkers has become an increasingly recognized strategy for managing training loads and reducing the risk of maladaptive responses, including prothrombotic states, in athletes. Traditional static assays of coagulation and platelet activity do not capture the dynamic interplay between blood flow, platelet aggregation, and coagulation cascade activation that occurs in vivo during physiological stress induced by training and competition. The Total Thrombus-Formation Analysis System (T-TAS), an automated microchip-based flow chamber device, allows for quantitative assessment of thrombus formation under simulated arterial flow conditions, integrating platelet and coagulation contributions to overall thrombogenicity [5,8]. Such technologies show promise in evaluating hemostatic capacity beyond conventional tests, although their application specifically in athletic populations is still an emerging field requiring further research [8].

Simultaneous monitoring of oxidative stress biomarkers—malondialdehyde (MDA), total antioxidant capacity (TAC), and DNA oxidation products such as 8-hydroxy-2′-deoxyguanosine (8-OHdG)—provides insight into redox balance and the cumulative physiological load experienced by athletes. Evidence from longitudinal athlete studies indicates that oxidative stress markers fluctuate throughout training cycles, reflecting both acute responses and chronic adaptations. Elevated levels of TBARSs (thiobarbituric acid-reactive substances) and protein carbonyls and changes in antioxidant enzyme activities have been observed during intensified training periods, suggesting that increased oxidative stress correlates with high physical load and transient muscle fatigue [60,61].

Regular biochemical monitoring, including oxidative stress, inflammatory markers, and muscle damage indicators, is an effective tool for optimizing training processes, injury prevention, and enhancing recovery efficiency [60]. The sports science literature supports the use of comprehensive biomarker profiles encompassing hormonal, inflammatory, immunological, and redox parameters to quantitatively determine cumulative fatigue and physiological adaptation, facilitating data-driven decision-making in training periodization [60].

Integration of T-TAS assessments with oxidative biomarkers allows for a multi-modality monitoring strategy that captures both hemostatic dynamics and redox status. This combined approach can detect early signs of imbalance before clinical symptoms or performance decrements occur, enabling timely adjustments to training intensity, recovery periods, and nutritional interventions. Such proactive monitoring is especially important during periods of high training load, competitive phases, or in athletes predisposed to hypercoagulable states [5,8,41,61].

From a mechanistic perspective, exercise-induced ROS production can influence platelet activation and endothelial function, which are key upstream events in thrombogenesis. Acute increases in oxidative stress may enhance platelet aggregation and modify coagulation pathways, transiently increasing thrombotic potential. Longitudinal data support the fact that without adequate recovery and adaptive antioxidant responses, cumulative oxidative stress may contribute to endothelial dysfunction and prothrombotic signaling pathways [41,59,60,61].

Despite promising methodologies, the literature identifies gaps in standardized protocols for T-TAS application and in the longitudinal integration of redox and hemostatic monitoring in athletes. Most studies have focused on clinical populations or acute exercise effects; dedicated research on elite athletes, including sport-specific demands and seasonal fluctuations, remains limited. Continued investigation combining advanced hemostatic assays with biomarkers of oxidative stress, inflammation, and recovery will be instrumental in establishing evidence-based guidelines for the prevention of prothrombotic states in physically active populations [5,8,41,60,61].

### 4.9. Literature Search Strategy and Study Selection

A systematic literature search was conducted to identify relevant original and review articles investigating the relationships between oxidative stress, platelet function, coagulation, thrombus formation under flow conditions, obesity, and exercise, with particular emphasis on studies employing microfluidic thrombus formation assays, including the Total Thrombus-Formation Analysis System (T-TAS).

The electronic databases PubMed/MEDLINE, Scopus, and Web of Science were systematically searched from database inception until 31 December 2025. The search strategy combined controlled vocabulary (MeSH terms) and free-text keywords related to oxidative stress, thrombosis, obesity, and hemostatic function. The following search terms and Boolean operators were used:

(“oxidative stress” OR “reactive oxygen species” OR “ROS” OR “lipid peroxidation” OR “malondialdehyde” OR “MDA” OR “8-hydroxy-2′-deoxyguanosine” OR 8-OHdG OR “total antioxidant capacity” OR TAC OR FRAP)

AND (“thrombosis” OR “thrombus formation” OR “platelet activation” OR “platelet aggregation” OR “hemostasis” OR “coagulation” OR “fibrinolysis”) AND (“obesity” OR overweight OR “metabolic syndrome” OR adiposity)

AND (“T-TAS” OR “Total Thrombus-Formation Analysis System” OR “microfluidic” OR “flow-based assay”)

Additional searches included combinations of the above terms with exercise, physical activity, training load, and recovery, in order to capture the literature relevant to exercise-induced oxidative and thrombotic responses.

The reference lists of all retrieved articles and relevant systematic reviews were manually screened to identify additional eligible publications. The gray literature, conference abstracts, editorials, and case reports were excluded.

#### 4.9.1. Eligibility Criteria

Studies were included if they met the following criteria:

1. Original experimental, observational, or clinical studies, as well as systematic reviews and meta-analyses.
2. Investigations reporting biomarkers of oxidative stress, antioxidant capacity, platelet function, coagulation, fibrinolysis, or flow-dependent thrombus formation.
3. Studies conducted in humans or relevant animal models of obesity, metabolic dysfunction, cardiovascular disease, or exercise physiology.
4. Articles published in peer-reviewed journals and available in English.

#### 4.9.2. Studies Were Excluded if They Met the Following Criteria:

1. Did not report oxidative stress or hemostatic outcomes.
2. Were limited to in vitro assays lacking physiological relevance.
3. Consisted solely of narrative opinions, editorials, or case reports.

#### 4.9.3. Study Selection and Data Extraction

Two independent reviewers screened titles and abstracts for relevance. Full texts of potentially eligible articles were subsequently evaluated for inclusion. Any discrepancies were resolved through discussion and consensus.

For each included study, data were extracted on study design, population characteristics, oxidative stress biomarkers, hemostatic parameters, methods used (including T-TAS, ROTEM, Multiplate, and conventional assays), and key findings. Extracted data were synthesized qualitatively due to substantial methodological heterogeneity.

#### 4.9.4. Study About Obesity and Oxidative Stress

Numerous studies have clearly demonstrated that obesity is associated with increased systemic oxidative stress, including elevated levels of lipid peroxidation products (MDA and F2-isoprostanes), oxidative DNA damage markers (8-OHdG), and protein carbonyls, and impaired total antioxidant capacity [1,2,3,4,5]. These alterations contribute to endothelial dysfunction, platelet hyperreactivity, impaired fibrinolysis, and chronic low-grade inflammation, all of which are recognized drivers of a prothrombotic phenotype in obesity [1,3,6].

Independent investigations using the T-TAS have demonstrated that oxidative and inflammatory conditions modulate flow-dependent thrombus formation, platelet adhesion, and fibrin-rich clot development, particularly in cardiometabolic disorders such as diabetes, atherosclerosis, and metabolic syndrome [7,8,9,10,11,12,13,14,15,16,17,18,19,20,21,22,23,24,25,26,27,28,29,30,31,32,33,34,35,36,37,38,39,40,41,42,43,44,45,46,47,48,49,50,51,52,53,54,55,56,57,58,59,60,61,62,63,64,65,66,67,68,69,70,71,72,73,74,75,76]. Although obesity itself was not always the primary inclusion criterion, these studies support the mechanistic link between redox imbalance and enhanced thrombotic potential under flow conditions, which is the central principle captured by the T-TAS.

Thus, the proposed relationship between oxidative stress biomarkers and T-TAS parameters in obesity is strongly supported by pathophysiological plausibility and converging indirect evidence but remains insufficiently explored in direct clinical studies. This represents a critical gap in current knowledge, highlighting the need for dedicated prospective studies integrating oxidative stress profiling with microfluidic thrombus formation assays in obese populations [73,74,75,76]. This is why future prospective studies integrating oxidative stress biomarkers with microfluidic thrombus formation assays such as the T-TAS in obese populations are urgently needed to establish direct clinical correlations and improve individualized thrombotic risk stratification.

## 5. Future Perspectives

Future research should focus on the integrated assessment of oxidative stress biomarkers and functional hemostatic parameters to improve risk stratification for obesity-associated venous and arterial thrombosis. The combination of biochemical markers of oxidative damage with dynamic, flow-based measurements obtained using the Total Thrombus-Formation Analysis System (T-TAS) offers a unique opportunity to capture both molecular and functional aspects of thrombus formation in a physiologically relevant setting. One promising direction involves the development of composite thrombo-oxidative risk scores that integrate oxidative stress markers (e.g., MDA, 8-OHdG, and TAC) with T-TAS-derived parameters such as area under the curve (AUC), thrombus initiation time, and occlusion dynamics. Such multidimensional models may outperform traditional coagulation assays in predicting thrombotic events in obese individuals. Additionally, future studies should explore the incorporation of transcriptomic and epigenetic data related to redox regulation, platelet activation, and fibrinolysis. Integrating gene expression profiles of antioxidant enzymes (e.g., SOD, GPX, and CAT), redox-sensitive transcription factors, and fibrinolytic regulators with T-TAS functional readouts may provide deeper mechanistic insight into obesity-driven thrombotic phenotypes. From a translational perspective, longitudinal and interventional studies are warranted to determine whether lifestyle modifications, weight loss strategies, and antioxidant-targeted therapies can favorably modulate both oxidative stress markers and T-TAS parameters. Such approaches could support the implementation of the T-TAS as a monitoring tool for therapeutic efficacy in cardiometabolic and thrombotic risk management.

Future studies should also consider incorporating platelet-related indices, including mean platelet volume (MPV), red cell distribution width (RDW), and platelet count, as well as derived parameters such as the MPV-to-platelet ratio (MPR) and RDW-to-platelet ratio (RPR). These indices have been shown to reflect platelet activation and reactivity, complementing T-TAS functional readouts and oxidative stress biomarkers, and may further refine thrombotic risk stratification in obesity-associated hypercoagulability according to [37].

This addition emphasizes the potential utility of standard hematological indices in combination with microfluidic flow-based assays like the T-TAS to better capture platelet-driven thrombotic phenotypes.

### 5.1. Limitations of the Total Thrombus-Formation Analysis System (T-TAS)

Despite its high physiological relevance and increasing application in experimental and clinical research, the Total Thrombus-Formation Analysis System (T-TAS) exhibits several important limitations that currently restrict its broader clinical implementation. These limitations primarily involve challenges related to standardization, cost, accessibility, and insufficient large-scale clinical validation.

#### 5.1.1. Standardization Challenges

One of the principal limitations of the T-TAS is the lack of comprehensive methodological standardization across laboratories. Thrombus formation under flow conditions is highly sensitive to pre-analytical variables, including blood collection technique, anticoagulant type, time from venipuncture to analysis, temperature control, and sample handling. Minor deviations in these factors may significantly influence platelet activation, coagulation kinetics, and thrombus stability, thereby affecting assay reproducibility and inter-study comparability.

Furthermore, no universally accepted reference ranges or diagnostic thresholds have yet been established for T-TAS parameters in different age groups, sexes, or disease populations. Variability related to chip type (PL-chip vs. AR-chip), shear rate selection, and analytical algorithms further complicates result interpretation. These issues underscore the urgent need for international consensus guidelines, standardized protocols, and inter-laboratory quality control programs to ensure reliability and reproducibility of T-TAS measurements.

#### 5.1.2. Economic Cost and Technical Complexity

The high cost of instrumentation, proprietary microfluidic cartridges, and consumables represents a major barrier to routine clinical use. Compared with conventional platelet function tests or viscoelastic assays, the T-TAS requires specialized equipment, trained technical staff, and continuous maintenance, which significantly increases operational costs. Additionally, the single-use nature of microfluidic chips further elevates the per-test cost, limiting feasibility for large-scale studies, repeated monitoring, and routine clinical screening.

Consequently, cost-effectiveness analyses remain limited, and economic constraints currently restrict T-TAS implementation mainly to research laboratories and highly specialized clinical centers.

#### 5.1.3. Limited Availability and Infrastructure Requirements

The availability of the T-TAS is restricted to selected academic and tertiary care institutions, particularly in Europe and East Asia, with limited access in many healthcare systems worldwide. The necessity for prompt sample processing after blood collection further limits feasibility in multicenter trials, epidemiological studies, and routine outpatient settings. Moreover, the absence of standardized training programs and certification pathways for laboratory personnel contributes to uneven methodological expertise and variable data quality across institutions.

#### 5.1.4. Gaps in Clinical Validation

Although the T-TAS provides a highly physiological assessment of thrombus formation under flow, robust prospective evidence linking T-TAS parameters to major clinical outcomes (e.g., myocardial infarction, ischemic stroke, and venous thromboembolism) remains limited. Most available studies are cross-sectional, involve relatively small cohorts, and focus primarily on surrogate laboratory endpoints rather than hard clinical events.

Consequently, clinically validated cut-off values for thrombotic risk stratification and treatment guidance have not yet been firmly established, which restricts routine clinical decision-making based on T-TAS results. Moreover, evidence regarding the utility of the T-TAS for longitudinal monitoring, therapy optimization, and individualized risk prediction remains insufficient, particularly in populations with obesity, metabolic syndrome, and chronic inflammatory conditions.

To fully realize the clinical potential of the T-TAS, future research should prioritize large-scale, multicenter prospective studies, aimed at validating the predictive value of T-TAS parameters for clinically relevant thrombotic outcomes. Additionally, efforts toward methodological harmonization, standard operating procedures, cost reduction strategies, and broader clinical accessibility will be essential to facilitate widespread adoption of this promising microfluidic technology.

## 6. The Lack of Fully Standardized Reference Ranges Currently Represents a Major Limitation for Routine Clinical Implementation of the T-TAS

We propose a stepwise and context-driven translational strategy that enables clinically meaningful use of the T-TAS while large-scale standardization efforts are ongoing.

First, the T-TAS should be implemented primarily as a comparative and longitudinal monitoring tool, rather than a standalone diagnostic assay. In this approach, each patient serves as their own internal reference, allowing detection of dynamic changes in thrombus formation parameters over time, particularly in response to training load, pharmacological interventions, metabolic fluctuations, or inflammatory states. Such longitudinal assessment reduces reliance on population-based reference intervals and enhances sensitivity to clinically relevant trends.

Second, the T-TAS can be incorporated as a complementary technique alongside established hemostatic assays, including ROTEM/TEG, conventional coagulation tests, and platelet aggregation methods. This multimodal strategy enables integrated interpretation of hemostatic function, improving diagnostic resolution in complex clinical scenarios such as obesity-associated hypercoagulability, metabolic syndrome, and exercise-induced thrombotic risk.

Third, implementation should initially focus on well-defined clinical contexts, including:

- Monitoring of antiplatelet and anticoagulant therapy;
- Risk stratification in cardiometabolic disorders;
- Evaluation of exercise-induced thrombotic risk;
- Assessment of prothrombotic tendencies in obesity and chronic inflammation.

Within these populations, local reference ranges and decision thresholds can be established using internal validation cohorts, enabling progressive standardization at the institutional level.

Finally, the development of large-scale, multicenter prospective studies and international registry-based initiatives, designed to generate robust population-based reference intervals, clinically validated cut-off values, and outcome-driven decision algorithms, are needed. Such coordinated efforts will be essential for the full clinical translation of the T-TAS.

Taken together, a phased translational model, combining longitudinal intra-individual monitoring, multimodal laboratory integration, and progressive multicenter validation, will allow for meaningful clinical deployment of the T-TAS despite current limitations in standardized reference ranges.

### Integration of Single-Cell Transcriptomics, Oxidative Stress Biomarkers, and Microfluidic Hemostatic Profiling

Recent advances in single-cell transcriptomics have fundamentally reshaped our understanding of platelet heterogeneity, revealing the existence of functionally distinct platelet subpopulations dynamically regulated by inflammatory, metabolic, and oxidative stress-related stimuli. These molecular phenotypes extend beyond classical concepts of platelet activation, highlighting diverse transcriptional programs associated with mitochondrial metabolism, hypoxia response, immune signaling, and procoagulant activity. In this context, oxidative stress emerges as a critical regulatory axis, linking systemic redox imbalance with platelet reprogramming, endothelial dysfunction, and dysregulated thrombo-inflammatory responses [77].

Single-cell transcriptomic analyses have demonstrated that platelet clusters characterized by enhanced oxidative phosphorylation, activation of coagulation cascades, and hypoxia-responsive gene signatures exhibit strong associations with disease severity and adverse clinical outcomes, particularly in inflammatory syndromes such as sepsis and COVID-19. These molecular signatures closely align with elevated circulating levels of oxidative stress biomarkers, including malondialdehyde (MDA), F2-isoprostanes, and 8-hydroxy-2′-deoxyguanosine (8-OHdG), which collectively reflect lipid, protein, and DNA oxidation. Such redox-driven transcriptional reprogramming promotes platelet hyperreactivity, accelerated thrombin generation, impaired fibrinolysis, and increased thrombus stability, thereby contributing to a systemic prothrombotic milieu [77].

Within this multidimensional framework, microfluidic flow-based assays such as the Total Thrombus-Formation Analysis System (T-TAS) provide a unique functional readout of the integrated effects of platelet activation, coagulation dynamics, and fibrin architecture under physiologically relevant shear conditions. Unlike conventional static assays, the T-TAS captures real-time thrombus formation, propagation, and occlusion kinetics, enabling the detection of subtle functional consequences arising from molecular alterations identified at the transcriptomic level. Consequently, integrating single-cell transcriptomic biomarkers with oxidative stress profiling and T-TAS-derived functional parameters offers a powerful, system-level approach to thrombotic risk stratification [77].

This integrated strategy holds particular promise in complex clinical settings characterized by chronic inflammation, metabolic dysregulation, and redox imbalance, such as obesity, metabolic syndrome, cardiovascular disease, and critical illness. By linking molecular signatures, biochemical indicators, and dynamic functional outcomes, this multidimensional approach may enable earlier detection of prothrombotic trajectories, improved patient stratification, and the development of personalized therapeutic strategies, ultimately advancing precision medicine in thrombo-inflammatory disorders [77].

## 7. Limitations

Several limitations should be acknowledged when interpreting studies integrating oxidative stress markers with T-TAS-based hemostatic assessment. First, oxidative stress biomarkers are often influenced by pre-analytical variables, including sample handling, storage conditions, and dietary factors, which may introduce variability and limit inter-study comparability. Standardization of analytical protocols remains a significant challenge.

Second, while the T-TAS provides a global and physiologically relevant assessment of thrombus formation under flow, it does not allow for the direct attribution of observed effects to specific molecular pathways or individual coagulation factors. Therefore, T-TAS results should be interpreted in conjunction with targeted biochemical and molecular analyses.

Third, most available data are derived from cross-sectional studies with relatively small sample sizes, limiting the ability to infer causality between oxidative stress and thrombotic risk in obesity. Prospective cohort studies and randomized controlled trials are necessary to validate the predictive value of combined oxidative stress and T-TAS parameters.

Finally, the cost and limited availability of T-TAS instrumentation may restrict its widespread use in routine clinical practice. Further efforts are required to establish standardized reference ranges and to determine cost-effectiveness before broader implementation can be recommended.

## 8. Conclusions

The T-TAS provides a unique, physiologically relevant assessment of thrombus formation under flow, integrating platelet function and coagulation in a way that static assays cannot. ROTEM offers robust global coagulation profiling, particularly useful in acute care settings. Multiplate excels in evaluating platelet response to agonists and monitoring antiplatelet therapy but lacks information on thrombus stability or flow-dependent interactions. Together, these tools are complementary, allowing for comprehensive evaluation of hemostatic function in both research and clinical contexts, with the T-TAS being particularly suitable for investigating flow-dependent prothrombotic phenotypes induced by obesity and oxidative stress. In summary, the T-TAS offers several methodological and conceptual advantages over thromboelastography in the assessment of obesity-associated thrombosis. Its flow-based design, heightened sensitivity to platelet function, and ability to model surface-dependent thrombus formation makes it particularly well suited for studying thrombotic mechanisms driven by oxidative stress, endothelial dysfunction, and metabolic dysregulation. While thromboelastography remains a valuable tool for global coagulation assessment, the T-TAS provides a more physiologically relevant platform for investigating the complex, flow-dependent nature of thrombosis in obesity.

## Figures and Tables

**Table 1:** Oxidative stress markers, hemostatic impact, and T-TAS readouts in obesity-associated thrombosis.

Category	Oxidative Stress Marker	Biological Significance	Potential Impact on Hemostasis	Relevant T-TAS Parameter(s)	Interpretation in Obesity-Associated Thrombosis
Lipid peroxidation	MDA/TBARS	End products of lipid peroxidation reflecting systemic oxidative stress	Promotes platelet activation and alters fibrin structure, demonstrated in flow-based assays	AUC (PL-chip, AR-chip); Occlusion time	Higher MDA levels correlate with increased thrombus formation and faster channel occlusion [5,6,7,8,9,10]
Lipid oxidation	F2-Isoprostanes	Stable markers of oxidative damage to membrane lipids	Induces endothelial dysfunction and enhances platelet reactivity, confirmed in experimental thrombosis models	AUC; Initial pressure increase	Elevated F2-isoprostanes indicate increased thrombotic potential under flow conditions [5,6,7,8,9,10,11,12,13,14,15,16,17,18,19,20,21,22,23,24,25,26,27,28,29,30,31,32,33,34,35,36,37,38,39,40,41,42,43,44,45,46,47,48,49,50,51,52,53,54,55,56,57,58,59,60,61,62]
DNA oxidative damage	8-OHdG	Marker of oxidative DNA damage	Activates pro-inflammatory and procoagulant pathways, supported by in vitro and in vivo studies	AUC; Time to thrombus initiation	Higher 8-OHdG levels reflect a systemic prothrombotic environment and endothelial dysfunction [5,6,7,8,9,10,11,12,13,14,15,16,17,18,19,20,21,22,23,24,25,26,27,28,29,30,31,32,33,34,35,36,37,38,39,40,41,42,43,44,45,46,47,48,49,50,51,52,53,54,55,56,57,58,59,60,61,62,63]
Protein oxidation	Protein carbonyls	Irreversible oxidative modification of plasma proteins	Impairs fibrinolysis and promotes formation of dense, lysis-resistant thrombi, observed in T-TAS assays	AUC (AR-chip); Pressure slope	Elevated protein carbonyls are linked to denser, more stable thrombi [5]
Antioxidant capacity	TAC/FRAP	Overall ability to neutralize reactive oxygen species	Protects against platelet hyperactivity and excessive coagulation, evidenced by delayed occlusion in flow assays	Delayed occlusion time; Reduced AUC	Lower TAC corresponds to enhanced thrombotic tendency and a procoagulant milieu [5,8]
Redox-regulated fibrinolysis	PAI-1	Inhibitor of plasminogen activation	Directly contributes to hypofibrinolysis and persistent clot formation, confirmed by prolonged pressure rise in T-TAS	Sustained pressure increase; High AUC	Elevated PAI-1 promotes stable, persistent thrombi under oxidative stress [5,6,7,8,9,10,11,12,13,14,15,16,17,18,19,20,21,22,23,24,25,26,27,28,29,30,31,32,33,34,35,36,37,38,39,40,41,42,43,44,45,46,47,48,49,50,51,52,53,54,55,56,57,58,59,60,61,62,63,64,65]

**Table 2:** Principal features, strengths, limitations, and sensitivity/specificity of T-TAS, ROTEM, and Multiplate (MEA). Legend for Table 2: T-TAS (Total Thrombus-Formation Analysis System) evaluates platelet adhesion, aggregation, and fibrin formation under controlled flow, providing physiological relevance to in vivo thrombus formation; ROTEM (Rotational Thromboelastometry) measures viscoelastic clot formation, providing global coagulation kinetics and clot strength, but lacks flow conditions and sensitivity to primary platelet hyperreactivity without adjunct platelet mapping; Multiplate (MEA, Multiple Electrode Aggregometry) assesses agonist-specific platelet aggregation in whole blood but does not provide information on thrombus stability, fibrin formation, or coagulation kinetics. Sensitivity/Specificity columns summarize evidence-based detection capabilities for antiplatelet therapy, coagulation disorders, and platelet function defects based on the current literature. Abbreviations: AUC—area under the curve (thrombus formation or aggregation); PL-chip/AR-chip—platelet or arachidonic acid microchips in T-TAS; DAPT—dual antiplatelet therapy.

Feature/System	T-TAS	ROTEM (+Platelet Mapping)	Multiplate (MEA)
Assay type	Microfluidic, flow-based thrombus formation	Viscoelastic clot formation (with optional platelet mapping)	Platelet aggregation (impedance-based)
Blood type	Whole blood	Whole blood	Whole blood
Flow conditions	Yes—controlled shear, physiological	No (static)	No (static)
Assesses platelets	Yes—adhesion and aggregation under flow	Indirectly via clot firmness (platelet contribution)	Yes—agonist-specific aggregation (ADP, AA, and collagen)
Assesses coagulation	Yes—integrated with thrombus formation	Yes—global clot kinetics and strength	No
Assesses fibrinolysis	Partial—via occlusion persistence	Yes—clot lysis parameters	No
Physiological relevance	High—mimics in vivo thrombus formation	Moderate—mechanical clot properties only	Low—agonist-specific platelet reactivity
Sensitivity to platelet inhibitors	High—flow-mediated inhibition detectable; AUC10/PL-chip cutoff < 260 discriminates antiplatelet therapy; sensitivity: DAPT 68–100%, aspirin alone ~68%	Limited—requires platelet mapping; sensitivity ~70–80% depending on protocol	High—agonist-dependent; ADP 85–90%, AA 70–100%
Specificity/clinical discrimination	High—differentiates DAPT vs controls; specificity 80–90% (AUC-based)	Moderate—detects coagulation factor effects; specificity ~85%	Moderate to high—identifies severe platelet defects; specificity for mild disorders <50%
Clinical utility	Thrombosis risk assessment; evaluation of platelet + coagulation under flow	Coagulopathy monitoring; transfusion guidance; anticoagulant effect quantification	Platelet function testing; monitoring antiplatelet therapy; prediction of high on-treatment platelet reactivity
Limitations	Requires standardization; limited large-cohort validation; less optimized for severe thrombocytopenia	No flow; less sensitive to primary platelet hyperreactivity; requires platelet mapping for antiplatelet detection	Does not measure thrombus stability or coagulation dynamics; limited for mild platelet dysfunction; no fibrinolysis assessment

**Table 3:** Comparative overview.

Feature	T-TAS PL-chip	ROTEM (+Platelet Mapping)	Multiplate (MEA)
Physiological relevance	High—mimics in vivo thrombus formation under flow	Moderate—mechanical clot properties only	Low—agonist-specific platelet reactivity
Sensitivity to antiplatelet therapy	Excellent—AUC10 < 260; discriminates DAPT vs. controls; 68–100% DAPT and aspirin ~68%	Low—aspirin ~86% and clopidogrel ~67% with mapping; standard ROTEM insensitive	High—ADP 85–90%; AA 70–100%
Specificity	Strong—reliably separates responders vs. non-responders to DAPT	Low—limited for platelet inhibition; better for fibrin/GPIIb/IIIa	Moderate to high—dependent on agonist and cut-off
Clinical utility	Thrombosis risk assessment; platelet + coagulation evaluation under flow	Coagulopathy monitoring; transfusion guidance; anticoagulant effect quantification	Platelet function testing; monitoring P2Y12/aspirin; high on-treatment reactivity prediction
Limitations	Requires standardization; population-dependent cut-offs; limited validation	No flow; insensitive without mapping; less sensitive than T-TAS/Multiplate	Limited to platelet aggregation; no fibrinolysis; reagent-dependent

**Table 4:** Comparison of oxidative stress responses and platelet function according to exercise modality. Legend: Endurance exercise (aerobic) is characterized by a moderate, gradually increasing ROS response, transient reduction in platelet function, and a shorter time to redox homeostasis. Regular aerobic training generally increases TAC and decreases lipid peroxidation (MDA) and DNA damage (8-OHdG) [36,37,38,39]. Resistance or explosive exercise generates an intense, short-lived oxidative stress with transient platelet hyperaggregation and prolonged return to homeostasis. Adaptive resistance training also increases TAC and decreases MDA, although 8-OHdG responses are more variable [40,41]. Training status modifies oxidative stress responses, with trained individuals showing smaller biomarker fluctuations compared with untrained individuals [42].

Parameter/Exercise Type	Endurance Exercise (Aerobic)	Resistance/Explosive Exercise
ROS Generation	Moderate, gradually increasing [38,39]	Intense, short-lived [40,41]
Lipid Peroxidation (MDA)	Decreased MDA after ≥12–24 weeks of training (−10% or more) [38,39]	Decreased MDA after adaptive training (~4.94 → ~3.90 μmol/L) [40]
DNA Damage (8-OHdG)	Reduced 8-OHdG compared with control groups [38,39]	Variable 8-OHdG responses, dependent on training status [40,41]
Total Antioxidant Capacity (TAC)	Increased TAC after regular aerobic training [38,39]	Increased TAC after adaptive resistance training [40,41]
Time to Redox Homeostasis	Shorter, dependent on exercise intensity	Longer and variable, dependent on volume and intensity
Effect of Training Status	Trained individuals exhibit smaller oxidative stress fluctuations than untrained individuals [42]	Trained individuals show less pronounced responses than untrained individuals, but differences are smaller than in aerobic exercise [42]

## Data Availability

No new data were created or analyzed in this study.

## Abbreviations

8-OHdG	8-Hydroxy-2′-deoxyguanosine
AUC	Area Under the Curve
APTT	Activated Partial Thromboplastin Time
AR-chip	Collagen Plus Tissue Factor-Coated Microchip (T-TAS)
BMI	Body Mass Index
CAT	Catalase
DNA	Deoxyribonucleic Acid
DOAC	Direct Oral Anticoagulant
FRAP	Ferric Reducing Ability of Plasma
GPX	Glutathione Peroxidase
GPIIb/IIIa	Glycoprotein IIb/IIIa
IL-6	Interleukin-6
MDA	Malondialdehyde
MCF	Maximum Clot Firmness
MEA	Multiple Electrode Aggregometry
Multiplate	Multiple Electrode Aggregometry System
NADPH	Nicotinamide Adenine Dinucleotide Phosphate
NF-κB	Nuclear Factor Kappa B
NO	Nitric Oxide
OS	Oxidative Stress
PAI-1	Plasminogen Activator Inhibitor-1
PL-chip	Collagen-Coated microchip (T-TAS)
PT	Prothrombin Time
ROS	Reactive Oxygen Species
ROTEM	Rotational Thromboelastometry
SOD	Superoxide Dismutase
TAC	Total Antioxidant Capacity
TBARS	Thiobarbituric Acid Reactive Substance
TEG	Thromboelastography
TF	Tissue Factor
T-TAS	Total Thrombus-Formation Analysis System
VTE	Venous Thromboembolism
